# Differences in mortality and causes of death between STEMI and NSTEMI in the early and late phases after acute myocardial infarction

**DOI:** 10.1371/journal.pone.0259268

**Published:** 2021-11-17

**Authors:** Yasuaki Takeji, Hiroki Shiomi, Takeshi Morimoto, Ko Yamamoto, Yukiko Matsumura-Nakano, Kazuya Nagao, Ryoji Taniguchi, Kyohei Yamaji, Tomohisa Tada, Eri Toda Kato, Yusuke Yoshikawa, Yuki Obayashi, Satoru Suwa, Moriaki Inoko, Natsuhiko Ehara, Toshihiro Tamura, Tomoya Onodera, Hiroki Watanabe, Mamoru Toyofuku, Kenji Nakatsuma, Hiroki Sakamoto, Kenji Ando, Yutaka Furukawa, Yukihito Sato, Yoshihisa Nakagawa, Kazushige Kadota, Takeshi Kimura, the CREDO-Kyoto AMI Registry Wave-2 Investigators

**Affiliations:** 1 Department of Cardiovascular Medicine, Graduate School of Medicine, Kyoto University, Kyoto, Japan; 2 Department of Clinical Epidemiology, Hyogo College of Medicine, Nishinomiya, Japan; 3 Department of Cardiovascular Medicine, Osaka Red Cross Hospital, Osaka, Japan; 4 Department of Cardiology, Hyogo Prefectural Amagasaki Hospital, Amagasaki, Japan; 5 Division of Cardiology, Kokura Memorial Hospital, Kitakyushu, Japan; 6 Department of Cardiology, Shizuoka General Hospital, Shizuoka, Japan; 7 Department of Cardiovascular Medicine, Juntendo University Shizuoka Hospital, Izunokuni, Japan; 8 Cardiovascular Center, The Tazuke Kofukai Medical Research Institute, Kitano Hospital, Osaka, Japan; 9 Department of Cardiovascular Medicine, Kobe City Medical Center General Hospital, Kobe, Japan; 10 Department of Cardiology, Tenri Hospital, Tenri, Japan; 11 Department of Cardiology, Shizuoka City Shizuoka Hospital, Shizuoka, Japan; 12 Department of Cardiology, Japanese Red Cross Wakayama Medical Center, Wakayama, Japan; 13 Department of Cardiology, Mitsubishi Kyoto Hospital, Kyoto, Japan; 14 Department of Cardiovascular Medicine, Shiga University of Medical Science Hospital, Otsu, Japan; 15 Department of Cardiology, Kurashiki Central Hospital, Kurashiki, Japan; Osaka University Graduate School of Medicine, JAPAN

## Abstract

**Background:**

The detailed causes of death in non–ST-segment–elevation myocardial infarction (NSTEMI) have not been adequately evaluated compared to those in ST-segment elevation myocardial infarction (STEMI).

**Methods:**

The study population was 6,228 AMI patients who underwent percutaneous coronary intervention (STEMI: 4,625 patients and NSTEMI: 1,603 patients). The primary outcome was all-cause death.

**Results:**

Within 6 months after AMI, the adjusted mortality risk was not significantly different between NSTEMI patients and STEMI patients (HR: 0.83, 95%CI: 0.67–1.03, P = 0.09). Regarding the causes of death within 6 months after AMI, mechanical complications more frequently occurred in STEMI patients than in NSTEMI patients, while proportions of post resuscitation status on arrival and heart failure were higher in in NSTEMI patients than in STEMI patients. Beyond 6 months after AMI, the adjusted mortality risk of NSTEMI relative to STEMI was not significantly different. (HR: 1.04, 95%CI: 0.90–1.20, P = 0.59). Regarding causes of death beyond 6 months after AMI, almost half of deaths were cardiovascular causes in both groups, and breakdown of causes of death was similar between NSTEMI and STEMI.

**Conclusion:**

The mortality risk within and beyond 6 months after AMI were not significantly different between STEMI patients and NSTEMI patients after adjusting confounders. Deaths due to post resuscitation status and heart failure were more frequent in NSTEMI within 6 months after AMI.

## Introduction

Primary percutaneous coronary intervention (PCI) in ST-segment elevation myocardial infarction (STEMI) has been widely performed worldwide and mortality of STEMI patients has been improved [1–5] Non ST-segment elevation myocardial infarction (NSTEMI) is different from STEMI in many aspects including pathophysiology and treatment [4, 6, 7]. Clinical outcomes of NSTEMI patients may also be different from those of STEMI patients. In-hospital mortality risk in NSTEMI patients was reported to be better or equivalent as compared with that in STEMI patients, while long-term mortality risk in NSTEMI patients was reported to be higher than in STEMI patients in real world practice [8–12]. However, the reasons for the higher mortality risk and the causes of death in NSTEMI patients have not been fully evaluated yet. We, therefore, sought to compare short- and long-term clinical outcomes and the causes of death between NSTEMI and STEMI in a large-scale Japanese cohort study.

## Materials and methods

### Study population

**Fig 1:**
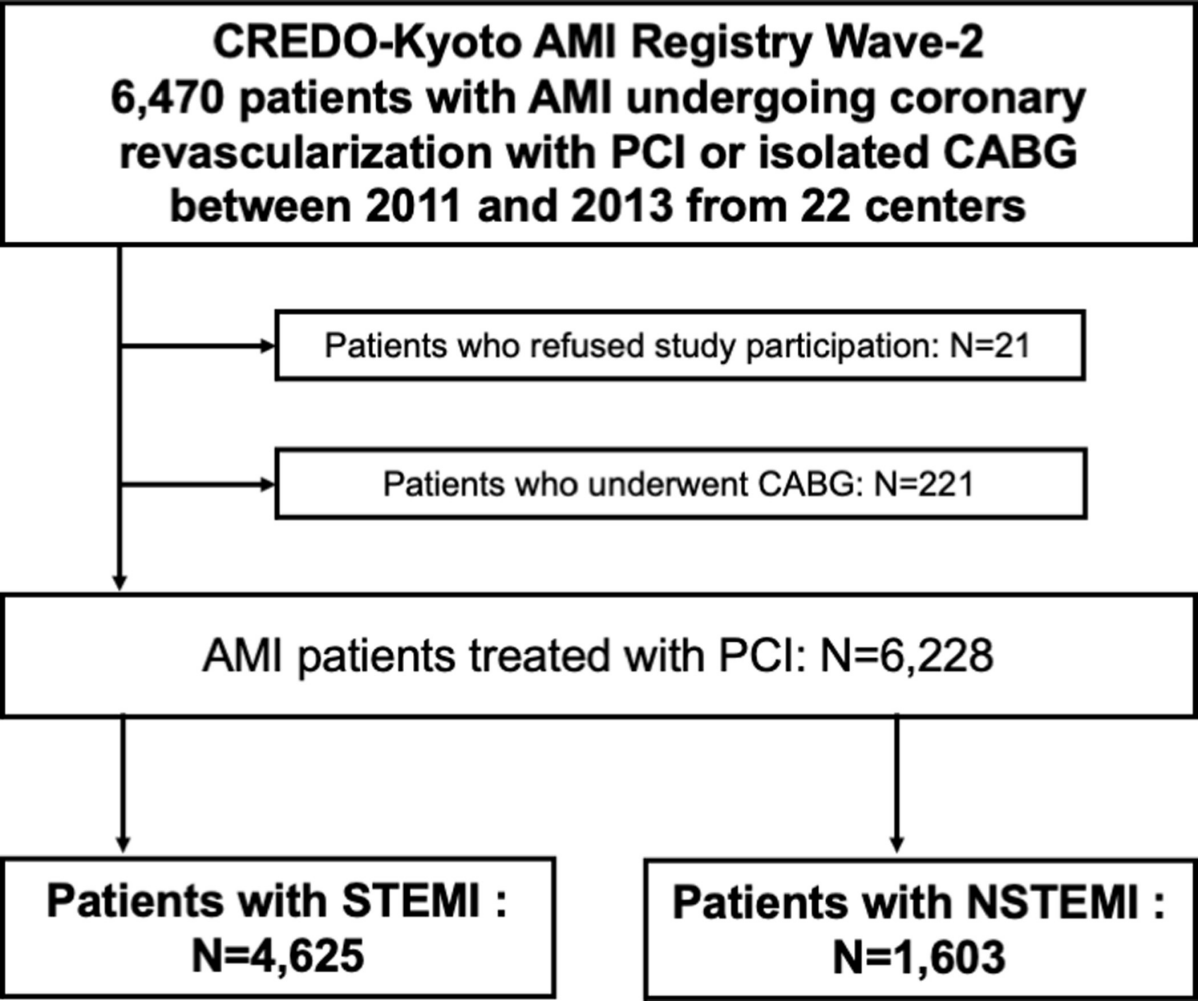
Study flowchart. CREDO-Kyoto = Coronary REvascularization Demonstrating Outcome study in Kyoto; AMI = acute myocardial infarction; PCI = percutaneous coronary intervention, CABG = coronary artery bypass grafting; NSTEMI = non-ST-segment elevation myocardial infarction; STEMI = ST-segment elevation myocardial infarction.

The Coronary REvascularization Demonstrating Outcome Study in Kyoto (CREDO-kyoto) acute myocardial infarction (AMI) Registry Wave-2 is a physician-initiated, non-company sponsored, multi-center registry enrolling 6,470 consecutive AMI patients who underwent coronary revascularization within 7 days of the onset of symptoms between January 2011 and December 2013 among 22 participating centers in Japan (S1 Text). In this study, the study population consisted of 6,228 AMI patients who underwent PCI after excluding those patients who refused study participation (N = 21) and those who underwent coroanry artery bypass grafting (CABG) (N = 221) (Fig 1). In the present study, we compared the baseline characteristics, clinical outcomes, and causes of death between those patients with STEMI and NSTEMI.

The relevant institutional review boards at all participating centers approved the study protocol, and we performed the study in accordance with the Declaration of Helsinki. Written informed consent for this study was waived because of the retrospective nature of the study; however, we excluded those patients who refused participation in the study when contacted at follow-up. This strategy is concordant with the guidelines of the Japanese Ministry of Health, Labor and Welfare.

### Definitions and clinical outcome measures

STEMI patients were defined as patients with electrocardiographic signs of ≥0.1 mV of ST-segment elevation in ≥2 limb leads or ≥0.2 mV in ≥2 contiguous precordial leads, accompanied by chest pain lasting at least 30 minutes or increased serum levels of cardiac biomarkers [13]. NSTEMI patients were defined as AMI other than STEMI, with elevating cardiac biomarkers, consisting of at least a value exceeding the upper reference limit for troponin, or >3× of the upper reference limit for creatine kinase MB (CK-MB). Experienced clinical research coordinators from the independent clinical research organization (Research Institute for Production Development, Kyoto, Japan; S2 Text) collected baseline clinical, angiographic and procedural characteristics from the hospital charts or hospital databases according to the pre-specified definitions.

The primary outcome measure of this study was all-cause death. The secondary outcome measures included cardiovascular death, non-cardiovascular death, myocardial infarction, stroke, hospitalization for heart failure (HF), major bleeding, target vessel revascularization, and any coronary revascularization. Death of unknown cause and any death during the index hospitalization for coronary revascularization were regarded as cardiac death. Cardiovascular death included cardiac death and other vascular death related to stroke, renal disease, and vascular disease. The definition of baseline characteristics and other endpoints were described at S3 Text. Death, myocardial infarction, stroke, and major bleeding events were adjudicated by the independent clinical event committee (S4 Text).

### Classification of causes of death

The detailed causes of cardiovascular death were classified into the followings; death related to the index AMI, recurrent AMI, sudden cardiac death or ventricular tachycardia (VT)/ventricular fibrillation (VF), heart failure, stroke, and other cardiovascular death. Furthermore, death related to the index AMI was sub-classified into cardiogenic shock, mechanical complication, anoxic brain damage, cardiopulmonary arrest (CPA) on arrival, and other causes during index hospitalization for AMI. The detailed causes of non-cardiovascular death were classified into malignancies, pulmonary disease, infection, gastrointestinal diseases, accident/trauma, and other non-cardiovascular death. To assess detailed causes of deaths, two physicians reviewed the patients’ records, and ascertained the causes of death independently. In case of disagreement, the third physician reviewed the patients’ records and discussed to reach a consensus about the causes of death.

### Statistical analyses

Continuous variables were expressed as mean ± standard deviation (SD) or median with interquartile range (IQR). Continuous variables were compared using the Student’s t-test or Wilcoxon rank sum test based on their distributions. Categorical variables are expressed as numbers and percentages and were compared using χ^2^ test. Cumulative incidence was estimated by the Kaplan-Meier method and differences were assessed with the log-rank test. To estimate the adjusted hazard ratios (HRs) and their 95% confidence intervals (CIs) of NSTEMI relative to STEMI for the outcome measures, we used the multivariable Cox proportional hazard models by incorporating the 28 clinically relevant factors listed in Table 1 in consistent with our previous report [13]. Continuous variables were dichotomized by clinically meaningful reference values to make proportional hazard assumptions robust and to be consistent with our previous reports [14]. Proportional hazard assumptions for the risk-adjusting variables were assessed on the plots of log (time) versus log [-log (survival)] stratified by the variable. The assumptions were verified to be acceptable for all the variables except for the primary variable (NSTEMI versus STEMI). A prior study also demonstrated that the primary variable did not meet the proportional hazard assumption.^6^ Therefore we conducted landmark analyses and estimated the adjusted HRs and their 95% CIs within and beyond 6 months after the index AMI. The missing values at baseline characteristics were described at S5 Text. All analyses were performed using R version 3.6.1 (R Foundation for Statistical Computing, Vienna, Austria). All reported P values were two-tailed, and P values less than 0.05 were considered statistically significant.

**Table 1:** Baseline characteristics comparing between NSTEMI and STEMI.

	NSTEMI	STEMI	P value
	(N = 1603)	(N = 4625)	
**(A) Clinical characteristics**			
Age (years)	70.5±11.5	68.8±12.6	<0.001
Age ≥75 years*	657 (41%)	1652 (36%)	<0.001
Men*	1214 (76%)	3459 (75%)	0.47
Body mass index (kg/m^2^)	23.8±3.6	23.6±3.6	0.30
Body mass index <25.0 kg/m^2^*	1077 (67%)	3207 (69%)	0.12
Hypertension*	1350 (84%)	3691 (80%)	<0.001
Diabetes mellitus*	636 (40%)	1614 (35%)	0.001
on insulin therapy	128 (8.0%)	259 (5.6%)	0.001
Current smoking*	450 (28%)	1676 (36%)	<0.001
Heart failure (current and/or prior)*	509 (32%)	1508 (33%)	0.55
LVEF (%)	56.3±13.4	53.8±12.3	<0.001
LVEF ≤40%	170 (12%)	580 (14%)	0.17
Prior PCI	348 (22%)	514 (11%)	<0.001
Prior CABG	64 (4.0%)	59 (1.3%)	<0.001
Prior myocardial infarction*	242 (15%)	417 (9.0%)	<0.001
Prior stroke (symptomatic)*	235 (15%)	505 (11%)	<0.001
Peripheral vascular disease*	105 (6.6%)	202 (4.4%)	0.001
eGFR<30mL/min/1.73m^2^, without hemodialysis*	122 (7.6%)	279 (6.0%)	0.03
Hemodialysis*	104 (6.5%)	119 (2.6%)	<0.001
eGFR <30 ml/min/1.73m^2^ or hemodialysis	226 (14%)	398 (8.6%)	<0.001
Atrial fibrillation*	179 (11%)	410 (8.9%)	0.008
Anemia (Hemoglobin <11.0g/dl) *	261 (16%)	505 (11%)	<0.001
Thrombocytopenia (Platelet <100×10^9^ /L) *	43 (2.7%)	93 (2.0%)	0.14
Chronic obstructive pulmonary disease*	68 (4.2%)	169 (3.7%)	0.33
Liver cirrhosis*	35 (2.2%)	99 (2.1%)	0.99
Malignancy*	189 (12%)	501 (11%)	0.31
**(B) Presentation**			
Transport pattern			<0.001
Direct admission	984 (61%)	2580 (56%)	
Inter-facility transfer	542 (34%)	1937 (42%)	
Others	77 (4.8%)	108 (2.3%)	
Systolic blood pressure	134±40	128±40	<0.001
Killip class III/IV	262 (16%)	882 (19%)	0.02
Cardiogenic shock*	180 (11%)	741 (16%)	<0.001
Cardiopulmonary arrest	60 (3.7%)	190 (4.1%)	0.57
Maximum creatine kinase	442 (163–1198)	1865 (788–3690)	<0.001
**(C) Angiographic characteristics**			
Multivessel disease*	991 (62%)	2560 (55%)	<0.001
Target of proximal LAD*	799 (50%)	2597 (56%)	<0.001
Target of unprotected left main coronary artery*	111 (6.9%)	213 (4.6%)	<0.001
Anterior wall infarction*	701 (44%)	2283 (49%)	<0.001
Infarct related artery location:			
Left anterior descending coronary artery	636 (40%)	2156 (47%)	<0.001
Left circumflex coronary artery	455 (28%)	470 (10%)	<0.001
Right coronary artery	450 (28%)	1875 (41%)	<0.001
Left main coronary artery	71 (4.4%)	137 (3.0%)	0.006
Coronary artery bypass graft	14 (0.9%)	23 (0.5%)	0.13
**(D) Procedural characteristics**			
Intra-aortic balloon pump use	209 (13%)	915 (20%)	<0.001
Percutaneous cardiopulmonary support use	46 (2.9%)	146 (3.2%)	0.63
Transradial approach	430 (27%)	733 (16%)	<0.001
Transfemoral approach	1055 (66%)	3640 (79%)	0.02
Intravascular ultrasound use for the culprit lesion	973 (61%)	2653 (57%)	0.24
Stent use for the culprit lesion	1454 (91%)	4241 (92%)	0.22
Stent type for the culprit lesion			<0.001
Bare metal stent	400 (28%)	1735 (41%)	
Drug-eluting stent	1054 (72%)	2506 (59%)	
Staged PCI	368 (23%)	1018 (22%)	0.45
**(E) Baseline Medications**			
Antiplatelet therapy			
Thienopyridine	1562 (97%)	4485 (97%)	0.38
Ticlopidine	79 (5.1%)	123 (2.7%)	<0.001
Clopidogrel	1454 (93%)	4304 (96%)	<0.001
Aspirin	1577 (98%)	4544 (98%)	0.82
Cilostazol	61 (3.8%)	114 (2.5%)	0.007
Statins*	1278 (80%)	3849 (83%)	0.06
Beta-blockers*	732 (46%)	2507 (54%)	<0.001
ACE inhibitors/ARB*	1117 (70%)	3525 (76%)	<0.001
Nitrates	356 (22%)	819 (18%)	<0.001
Calcium channel blockers*	587 (37%)	941 (20%)	<0.001
Nicorandil	324 (20%)	939 (20%)	0.97
Oral anticoagulant	174 (11%)	613 (13%)	0.01
Warfarin	154 (9.6%)	553 (12%)	0.01
Direct oral anticoagulant	21 (1.3%)	60 (1.3%)	1.00
Proton pump inhibitors or Histamine type-2 receptor blockers *	1304 (81%)	3961 (86%)	<0.001
Proton pump inhibitors	1089 (68%)	3431 (74%)	<0.001
Histamine type-2 receptor blockers	224 (14%)	544 (12%)	0.02

*Risk-adjusting variables for the Cox proportional hazard models.

STEMI = ST-segment elevation myocardial infarction; NSTEMI = Non ST-segment elevation myocardial infarction; LVEF = left ventricular ejection fraction; PCI = percutaneous coronary intervention; CABG = coronary artery bypass grafting; eGFR = estimated glomerular filtration rate; LAD = left anterior descending artery; PCI = percutaneous coronary intervention; ACE inhibitor/ARB = angiotensin-converting enzyme inhibitor/angiotensin receptor blocker.

## Results

### Study population and baseline characteristics in NSTEMI and STEMI

Among the study population of 6,228 AMI patients who underwent PCI, there were 4,625 patients with STEMI, and 1603 patients with NSTEMI. As compared with STEMI patients, NSTEMI patients were older, and more often had comorbidities such as hypertension, diabetes, severe renal dysfunction, peripheral vascular disease, and atrial fibrillation. The NSTEMI group also included more patients with prior myocardial infarction, prior coronary revascularization and prior stroke than the STEMI group (Table 1). However, the proportion of patients complicated by cardiogenic shock was significantly lower in the NSTEMI group than in the STEMI group, and infarct size estimated by maximum creatinine kinase (CK) was significantly smaller in the NSTEMI group than in the STEMI group (S1 Fig).

NSTEMI patients more often had their culprit lesions in left main coronary artery and left circumflex coronary artery, and more often had multivessel disease than STEMI patients. Regarding procedural characteristics, transradial approach and drug-eluting stents (DES) were more often used in the NSTEMI group than in the STEMI group.

In terms of medications at hospital discharge, STEMI patients more often received guideline-recommended medical therapy such as statins, beta-blockers, angiotensin-converting enzyme inhibitors/angiotensin receptor blockers than NSTEMI patients (Table 1).

### Long-term clinical outcomes in NSTEMI and STEMI

Median follow-up duration was 5.5 years (IQR, 3.6–6.6 years). Complete 1-, 3-, and 5-year follow-up information was obtained in 96.5%, 93.6%, and 83.1% of patients.

**Fig 2:**
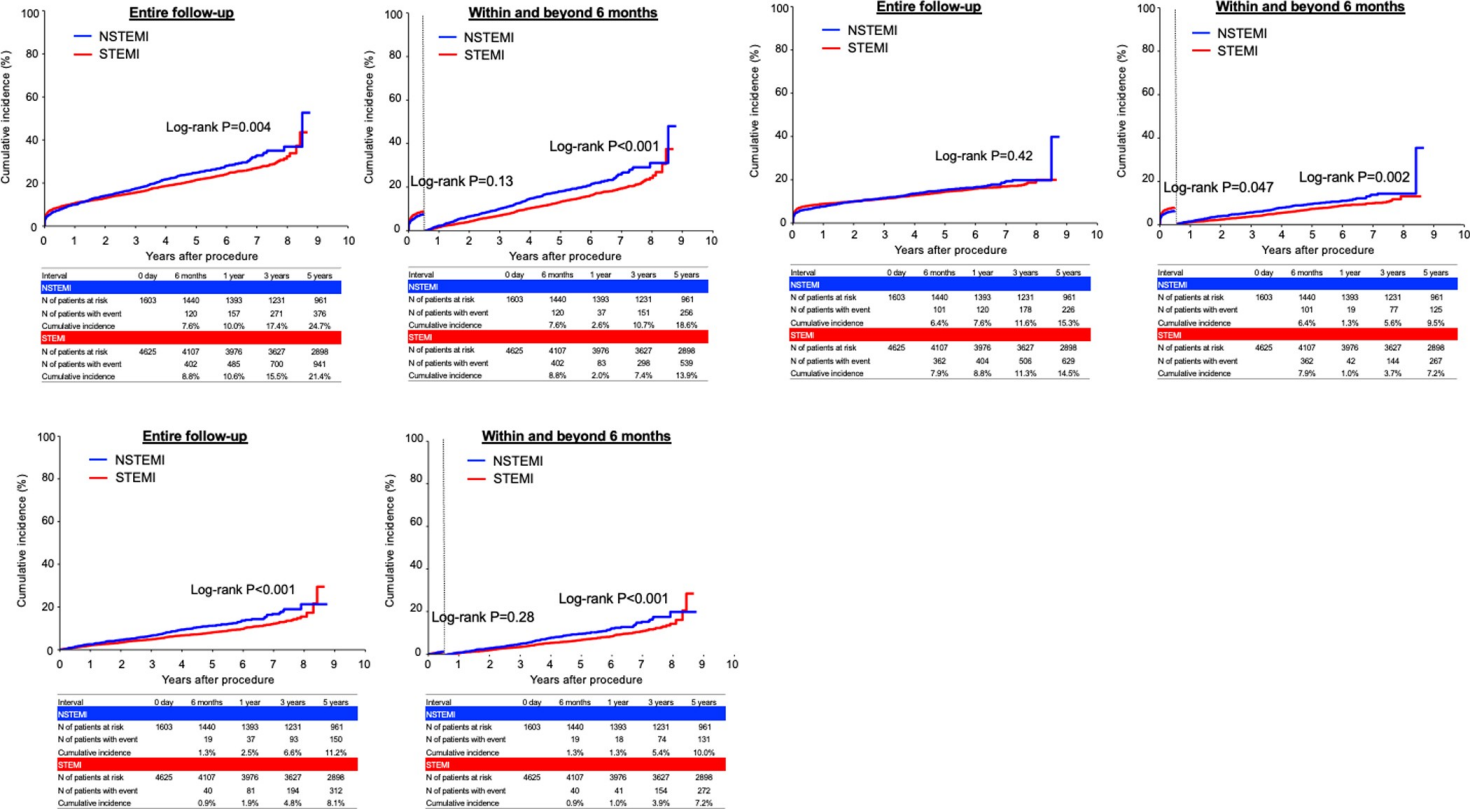
Kaplan-Meier curves for mortality outcomes comparing between NSTEMI and STEMI. (A) all-cause death, (B) cardiovascular death, and (C) non-cardiovascular death. NSTEMI = non-ST-segment elevation myocardial infarction; STEMI = ST-segment elevation myocardial infarction.

The cumulative 5-year incidence of all-cause death was significantly higher in the NSTEMI group than in the STEMI group (24.7% versus 21.4%, P = 0.004) (Fig 2). The cumulative 5-year incidence of cardiovascular death was not significantly different between the 2 groups (15.3% versus 14.5%, P = 0.42), while the cumulative 5-year incidence of non-cardiovascular death was significantly higher in the NSTEMI group than in the STEMI group (11.2% versus 8.1%, P<0.001) (Fig 2). The cumulative 5-year incidences of myocardial infarction, hospitalization for heart failure, target vessel revascularization, and any coronary revascularization were significantly higher in the NSTEMI group than in the STEMI group (9.1% versus 6.7%, P = 0.002, 13.0% versus 9.6%, P<0.001, 24.5% versus 21.4%, P = 0.04, and 33.3% versus 29.4%, P = 0.02, respectively) (S2 Fig). The cumulative 5-year incidences of stroke and major bleeding were not significantly different between the 2 groups (7.5% versus 7.5%, P = 0.93, and 23.5% versus 21.5%, P = 0.35, respectively) (S2 Fig).

### Difference of mortality and causes of deaths between NSTEMI and STEMI in acute phase (within 6 months after AMI)

The cumulative incidence of all-cause death within 6 months after AMI was not significantly different between the NSTEMI and STEMI groups (7.6% versus 8.8%, log-rank P = 0.13) (Fig 2, and Table 2). The adjusted risk for all-cause death was not significantly different between NSTEMI group and STEMI group (adjusted HR: 0.83, 95%CI: 0.67–1.03, P = 0.09) (Table 2). The cumulative incidence of cardiovascular death within 6 months were significantly lower in the NSTEMI group than in the STEMI group, while the adjusted risk for cardiovascular death within 6 months was not significantly different between the NSTEMI group and the STEMI group (6.4% versus 7.9%, log-rank P = 0.047, and adjusted HR: 0.82, 95%CI: 0.65–1.03, P = 0.09) (Fig 2 and Table 2). The adjusted risks of NSTEMI relative to STEMI for stroke, hospitalization for HF tended to be lower but statistically not significant (Table 2). The adjusted risks for major bleeding was significantly lower in the NSTEMI group than in the STEMI group (adjusted HR: 0.81, 95%CI: 0.68–0.96, P = 0.01) (Table 2).

**Table 2:** Clinical outcomes comparing between NSTEMI and STEMI within and beyond 6 months.

Endpoints	NSTEMI		STEMI		Crude HR	P value	Adjusted HR	P value
	
	N of patients with event				(95%CI)		(95%CI)	
	(Cumulative incidence)							
All-cause death								
Within 6 months	120	(7.6%)	402	(8.8%)	0.86(0.70–1.05)	0.13	0.83(0.67–1.03)	0.09
Beyond 6 months	321	(18.6%)	694	(13.9%)	1.37(1.20–1.56)	<0.001	1.04(0.90–1.20)	0.59
Cardiovascular death								
Within 6 months	101	(6.4%)	362	(7.9%)	0.80(0.64–1.00)	0.047	0.82(0.65–1.03)	0.09
Beyond 6 months	151	(9.5%)	329	(7.2%)	1.35(1.12–1.64)	0.002	0.94(0.77–1.15)	0.55
Non-cardiovascular death								
Within 6 months	19	(1.3%)	40	(0.9%)	1.35(0.78–2.34)	0.28	-	-
Beyond 6 months	170	(10.0%)	365	(7.2%)	1.38(1.15–1.66)	0.001	1.13(0.93–1.37)	0.21
Myocardial infarction								
Within 6 months	41	(2.7%)	132	(3.0%)	0.89(0.62–1.26)	0.501	0.75(0.52–1.08)	0.12
Beyond 6 months	102	(6.5%)	174	(3.8%)	1.76(1.38–2.25)	<0.001	1.38(1.06–1.78)	0.02
Stroke								
Within 6 months	37	(2.4%)	120	(2.7%)	0.88(0.61–1.28)	0.51	0.71(0.48–1.04)	0.08
Beyond 6 months	80	(5.3%)	224	(4.9%)	1.05(0.81–1.35)	0.71	0.83(0.63–1.08)	0.16
Hospitalization for heart failure								
Within 6 months	54	(3.6%)	145	(3.4%)	1.06(0.77–1.45)	0.72	0.75(0.54–1.04)	0.08
Beyond 6 months	156	(9.7%)	291	(6.4%)	1.58(1.30–1.92)	<0.001	1.15(0.94–1.42)	0.19
Major bleeding								
Within 6 months	191	(12.1%)	638	(14.1%)	0.85(0.72–1.00)	0.052	0.81(0.68–0.96)	0.01
Beyond 6 months	177	(12.9%)	359	(8.6%)	1.43(1.20–1.71)	<0.001	1.16(0.96–1.40)	0.13
Target vessel revascularization								
Within 6 months	107	(7.1%)	305	(7.1%)	1.00(0.80–1.25)	0.99	0.84(0.67–1.06)	0.14
Beyond 6 months	252	(18.7%)	610	(15.4%)	1.21(1.05–1.4)	0.01	1.16(0.99–1.35)	0.07
Any coronary revascularization								
Within 6 months	139	(9.3%)	404	(9.4%)	0.98(0.81–1.19)	0.84	0.84(0.69–1.03)	0.09
Beyond 6 months	350	(26.5%)	853	(22.2%)	1.21(1.07–1.37)	0.003	1.14(1.00–1.30)	0.053

Number of patients with event beyond 6 months was counted until the end of follow-up.

Cumulative incidence was presented at 6-month for within 6 months, and at 5-year for beyond 6 months.

The risk of NSTEMI relative to STEMI was expressed as HR with 95%CI. The risk-adjusting variables in the multivariate Cox proportional hazard models were indicated in Table 1.

STEMI = ST-segment elevation myocardial infarction; NSTEMI = Non ST-segment elevation myocardial infarction; HR = hazard ratio; CI = confidence interval.

**Fig 3:**
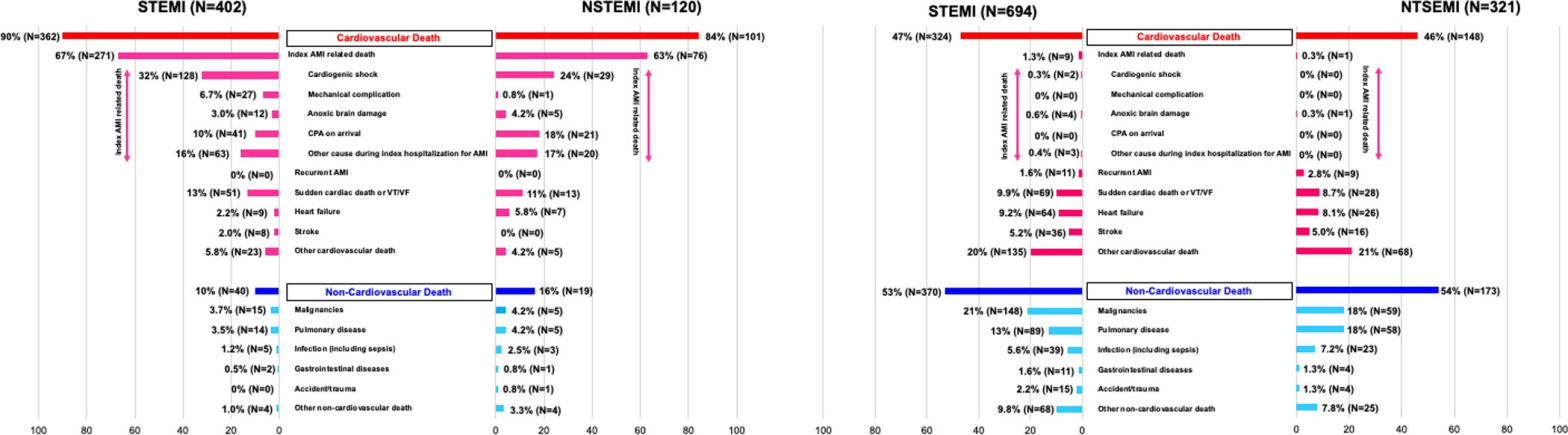
The detailed causes of deaths after NSTEMI and STEMI (A) in the early phase (within 6 months), and (B) in the late phase (beyond 6 months). STEMI = ST-segment elevation myocardial infarction; NSTEMI = Non ST-segment elevation myocardial infarction; VT = ventricular tachycardia; VF = ventricular fibrillation; CPA = cardiopulmonary arrest; AMI = acute myocardial infarction.

Regarding the detailed causes of cardiovascular death, approximately two thirds of deaths were related to the index AMI event in both NSTEMI and STEMI (Fig 3). Among the index AMI related death, mechanical complications (including ventricular septal perforation and myocardial rupture) occurred more frequently in the STEMI group than in the NSTEMI group (6.7% [N = 27], and 0.8% [N = 1], respectively), and the proportion of death due to cardiogenic shock was numerically higher in the STEMI group than in the NSTEMI group (32% [N = 128] and 24% [N = 29], respectively). However, the proportion of death due to post resuscitation status after CPA on arrival was greater in the NSTEMI group than in the STEMI group (18% [N = 21], and 10% [N = 41], respectively). Among 21 deaths due to post resuscitation status after CPA on arrival in the NSTEMI group, 15 patients had totally occluded culprit lesions on angiography. The proportion of death due to HF within 6 months after AMI was also higher in the NSTEMI group than in the STEMI group (5.8% [N = 7], and 2.2% [N = 9], respectively) (Fig 3).

### Difference of mortality and causes of deaths between NSTEMI and STEMI in late phase (beyond 6 months after AMI)

Beyond 6 months after AMI, the cumulative incidences of all-cause death, cardiovascular death, and non-cardiovascular death were significantly higher in the NSTEMI group than in the STEMI group (18.6% versus 13.9%, P<0.001, and 9.5% versus 7.2%, P = 0.002, and 10% versus 7.2%, P = 0.001, respectively). However, after adjusting for confounders, the risk of NSTEMI relative to STEMI was no longer significant for all-cause death, cardiovascular death, and non-cardiovascular death (adjusted HR: 1.04, 95%CI: 0.90–1.20, P = 0.59, and adjusted HR: 0.94, 95%CI: 0.77–1.15, P = 0.55, and adjusted HR: 1.13, 95%CI: 0.93–1.37, P = 0.21, respectively) (Table 2). The risk of NSTEMI relative to STEMI was significantly higher for myocardial infarction beyond 6 months (Table 2).

In both the NSTEMI and STEMI groups, approximately half of deaths were accounted for cardiovascular death (N = 148/321 [46%], and N = 324/694 [47%], respectively) (Fig 3B). Detailed causes of cardiovascular and non-cardiovascular deaths beyond 6 months after AMI were similar between the NSTEMI and STEMI groups (Fig 3).

## Discussion

The main findings of this study were as follows; 1) The mortality risk within 6 months after AMI was not significantly different between STEMI patients and NSTEMI patients; 2) Within 6 months after AMI, deaths due to post resuscitation status and HF were more frequent in NSTEMI patients than in STEMI patients, while deaths due to mechanical complication and cardiogenic shock were more frequent in STEMI patients than in NSTEMI patients; 3) The mortality risk of NSTEMI relative to STEMI beyond 6 months after AMI was no longer significant after adjusting confounders; 4) Approximately half of deaths beyond 6 months were cardiovascular death in both STEMI and NSTEMI patients, and detailed causes of deaths were similar in NSTEMI and STEMI patients.

Several previous studies reported that long-term prognosis in NSTEMI patients was worse than that in STEMI patients despite of smaller infarct size [8–12]. In this study, adjusted risk of NSTEMI relative to STEMI for long-term mortality was neutral. Discrepancy among studies including our study might be largely explained the heterogenous NSTEMI population and different follow-up duration among studies. After widespread use of troponin, especially high-sensitive troponin, the paradigm shift occurred in the concept of NSTEMI, which included many low risk patients who were formerly diagnosed as unstable angina.

Consistent with our study results, most previous studies reported that mortality risk of NSTEMI in acute phase of AMI was equivalent or slightly lower than that of STEMI, but the mortality risk of STEMI patients relative to NSTEMI patients attenuated overtime, because long-term mortality risk of NSTEMI beyond acute phase of AMI was higher than that of STEMI due to high risk patients’ backgrounds in NSTEMI patients such as older age and multiple comorbidities [4, 6, 7].

Even within 6 months after AMI, the absolute difference of mortality rate was only 1.2% (7.6% versus 8.8%), indicating that NSTEMI patients did not mean low risk population, but included high-risk patients in the heterogenous population. As mentioned above, NSTEMI patients were generally older and had more comorbidity, and more complex anatomy such as left main disease and multivessel disease than STEMI patients. The proportion of left main coronary artery and left circumflex coronary artery as a culprit lesion was significantly higher in NSTEMI patients than in STEMI patients in this study, suggesting that NSETMI population included high-risk subset of patients who presented “non-ST elevation” electrocardiographic findings, but had a “STEMI like” hemodynamic consequence during evolving myocardial infarction. The recent study reported the favorable clinical outcome of shorter door to balloon time even in NSTEMI population, suggesting the importance of appropriate risk stratification and timely reperfusion therapy in NSTEMI population [15].

According to the findings of this study results, the detailed causes of deaths in acute phase of AMI were somewhat different between STEMI and NSTEMI. Among those differences, it was worth noting that the proportion of deaths of post resuscitation status after CPA on arrival was higher in NSTEMI patients than in STEMI patients. Previous studies reported that ECG findings at the time of recovery of spontaneous circulation often did not show typical changes including ST segment elevation, and, therefore, clinical guidelines recommend coronary angiography unless other causes of cardiac arrest are identified and patients are comatose [16–18]. Actually, in this study, more than 70% of NSTEMI patients with death due to post resuscitation status after CPA on arrival had totally occluded culprit lesions on angiography. On the other hand, the proportion of mechanical complication such as ventricular septal perforation and cardiac rupture in this study was higher in STEMI patients than in NSTEMI patients. This could be explained by the angiographic characteristics. A prior study suggested that left anterior descending (LAD) artery and right coronary artery (RCA) were the vast majority of infarct related lesion for patients with ventricular septal perforation, and another study demonstrated single vessel disease was the risk factor for cardiac rupture [19, 20]. In this study, more patients with STEMI had LAD and RCA as infarct related artery than those with NSTEMI, and more patients with STEMI had single vessel disease compared with those with NSTEMI.

Beyond 6 months after AMI in this study, the mortality risk was not different between NSTEMI and STEMI. Among deaths during long-term follow-up, approximately half of deaths were cardiovascular death, and detailed caused of deaths were similar between NSTEMI and STEMI, indicating that secondary prevention and appropriate medical treatment is important in not only STEMI patients but also in NSTEMI patients. Considering high risk feature of NSTEMI patients with elderly and many comorbidities, further studies investigating appropriate management of NSTEMI patients might be needed.

### Limitations

There are several limitations of this study. First, the current study was an observational study with its limitations inherent to observational study design, such as selection bias and unmeasured confounders. Second, the specific landmark point of 6 months was not prespecified, but visually assessed by viewing the entire follow-up results in consistent with our previous report. Finally, we included only NSTEMI patients who underwent coronary revascularization. NSTEMI patients who were selected conservative therapy and who were contraindication for intervention were excluded, therefore the prognosis of NSTEMI patients in this study might be different from that of the entire NSTEMI population in daily clinical practice.

## Conclusions

The mortality risk within and beyond 6 months after AMI were not significantly different between STEMI patients and NSTEMI patients after adjusting confounders. Deaths due to post resuscitation status and heart failure were more frequent in NSTEMI within 6 months after AMI.

## Supporting information

S1 Fig Distribution of creatine kinase in patients with NSTEMI and STEMI. (DOCX)

S2 Fig Kaplan-Meier curves for other clinical outcomes comparing between NSTEMI and STEMI. (DOCX)

S1 Text List of participating centers and investigators in the CREDO-Kyoto AMI Registry Wave-2. (DOCX)

S2 Text List of clinical research coordinators in the CREDO-Kyoto AMI Registry Wave-2. (DOCX)

S3 Text Definition of baseline characteristics and endpoints. (DOCX)

S4 Text List of the clinical event committee members in the CREDO-Kyoto AMI Registry Wave-2. (DOCX)

S5 Text Missing values at baseline characteristics. (DOCX)
