# Supplementary material for: Differences in mortality and causes of death between STEMI and NSTEMI in the early and late phases after acute myocardial infarction
Source: PLoS One. 2021 Nov 17;16(11):e0259268. doi: 10.1371/journal.pone.0259268 (PMC8598015; doi:10.1371/journal.pone.0259268)
Supplement: S1 Fig — (DOCX) [file pone.0259268.s001.docx]

**S1 Fig. Distribution of creatine kinase in patients with NSTEMI and STEMI**

(A) in the entire cohort, (B) in patients who died within 6 months after AMI, and (C) in patients who died during the entire follow-up period.
