# Supplementary material for: Differences in mortality and causes of death between STEMI and NSTEMI in the early and late phases after acute myocardial infarction
Source: PLoS One. 2021 Nov 17;16(11):e0259268. doi: 10.1371/journal.pone.0259268 (PMC8598015; doi:10.1371/journal.pone.0259268)
Supplement: S2 Fig — (DOCX) [file pone.0259268.s002.docx]

**S2 Fig. Kaplan-Meier curves for other clinical outcomes comparing between NSTEMI and STEMI**

(A) myocardial infarction, (B) stroke, (C) heart failure hospitalization, (D) major bleeding, (E) target vessel revascularization, and (F) any coronary revascularization.
