# Supplementary material for: Differences in mortality and causes of death between STEMI and NSTEMI in the early and late phases after acute myocardial infarction
Source: PLoS One. 2021 Nov 17;16(11):e0259268. doi: 10.1371/journal.pone.0259268 (PMC8598015; doi:10.1371/journal.pone.0259268)
Supplement: S1 Text — (DOCX) [file pone.0259268.s003.docx]

**S1 Text. List of participating centers and investigators in the CREDO-Kyoto AMI Registry Wave-2**

**Cardiology**

Kyoto University Hospital: Takeshi Kimura, Hiroki Shiomi

Kishiwada City Hospital: Mitsuo Matsuda, Takashi Uegaito

Tenri Hospital: Toshihiro Tamura

Hyogo Prefectural Amagasaki General Medical Center: Yukihito Sato, Ryoji Taniguchi

Kitano Hospital: Moriaki Inoko

Koto Memorial Hospital: Tomoyuki Murakami, Teruki Takeda

Kokura Memorial Hospital: Kenji Ando, Takenori Domei

Kindai University Nara Hospital: Manabu Shirotani

Kobe City Medical Center General Hospital: Yutaka Furukawa, Natsuhiko Ehara

Kobe City Nishi-Kobe Medical Center: Hiroshi Eizawa

Kansai Denryoku Hospital: Katsuhisa Ishii, Eiji Tada

Osaka Red Cross Hospital: Masaru Tanaka, Tsukasa Inada

Shizuoka City Shizuoka　Hospital: Tomoya Onodera, Ryuzo Nawada

Hamamatsu Rosai Hospital: Eiji Shinoda, Miho Yamada

Shiga University of Medical Science Hospital: Takashi Yamamoto, Hiroshi Sakai

Japanese Red Cross Wakayama Medical Center: Takashi Tamura, Mamoru Toyofuku

Shimabara Hospital: Mamoru Takahashi

Shizuoka General Hospital: Hiroki Sakamoto, Tomohisa Tada

Kurashiki Central Hospital: Kazushige Kadota, Takeshi Tada

Mitsubishi Kyoto Hospital: Shinji Miki, Kazuhisa Kaneda

Shimada Municipal Hospital: Takeshi Aoyama

Juntendo University Shizuoka Hospital: Satoru Suwa

**Cardiovascular Surgery**

Kyoto University Hospital: Kenji Minatoya, Kazuhiro Yamazaki

Kishiwada City Hospital: Tatsuya Ogawa

Tenri Hospital: Atsushi Iwakura

Hyogo Prefectural Amagasaki General Medical Center: Nobuhisa Ohno

Kitano Hospital: Michiya Hanyu

Kokura Memorial Hospital: Yoshiharu Soga, Akira Marui

Kindai University Nara Hospital: Nobushige Tamura

Kobe City Medical Center General Hospital: Tadaaki Koyama

Osaka Red Cross Hospital: Shogo Nakayama

Shizuoka City Shizuoka　Hospital: Fumio Yamazaki, Yasuhiko Terai

Hamamatsu Rosai Hospital: Junichiro Nishizawa

Japanese Red Cross Wakayama Medical Center: Naoki Kanemitsu, Hiroyuki Hara

Shizuoka General Hospital: Hiroshi Tsuneyoshi

Kurashiki Central Hospital: Tatsuhiko Komiya

Mitsubishi Kyoto Hospital: Jiro Esaki

Juntendo University Shizuoka Hospital: Keiichi Tambara
