# Supplementary material for: Differences in mortality and causes of death between STEMI and NSTEMI in the early and late phases after acute myocardial infarction
Source: PLoS One. 2021 Nov 17;16(11):e0259268. doi: 10.1371/journal.pone.0259268 (PMC8598015; doi:10.1371/journal.pone.0259268)
Supplement: S2 Text — (DOCX) [file pone.0259268.s004.docx]

**S2 Text. List of clinical research coordinators in the CREDO-Kyoto AMI Registry Wave-2**

Research Institute for Production Development

Sakiko Arimura, Yumika Fujino, Miya Hanazawa, Chikako Hibi, Risa Kato, Yui Kinoshita, Kumiko Kitagawa, Masayo Kitamura, Takahiro Kuwahara, Satoko Nishida, Naoko Okamoto, Yuki Sato, Saori Tezuka, Marina Tsuda, Miyuki Tsumori, Misato Yamauchi, Itsuki Yamazaki
