# Supplementary material for: Differences in mortality and causes of death between STEMI and NSTEMI in the early and late phases after acute myocardial infarction
Source: PLoS One. 2021 Nov 17;16(11):e0259268. doi: 10.1371/journal.pone.0259268 (PMC8598015; doi:10.1371/journal.pone.0259268)
Supplement: S3 Text — (DOCX) [file pone.0259268.s005.docx]

**S3 Text. Definition of baseline characteristics and endpoints**

Baseline clinical characteristics, such as hypertension, current smoking, heart failure, prior myocardial infarction, atrial fibrillation, and chronic obstructive pulmonary disease were regarded as present when these diagnoses were recorded in hospital charts. Diabetes mellitus was defined as treatment with oral hypoglycemic agents or insulin, prior clinical diagnosis of diabetes, glycated hemoglobin level ≥6.5 %, or non-fasting blood glucose level ≥200 mg/dL. Prior stroke was defined as ischemic or hemorrhagic stroke with neurological symptoms lasting >24 hours. Peripheral vascular disease was regarded as present when carotid, aortic, or other peripheral vascular diseases were being treated or scheduled for surgical or endovascular interventions. Renal function was expressed as estimated glomerular filtration rate (eGFR) calculated by the Modification of Diet in Renal Disease formula modified for Japanese patients [1].

Myocardial infarction and stent thrombosis were adjudicated according to the Academic Research Consortium (ARC) definition [2]. Stroke was defined as ischemic or hemorrhagic stroke either requiring hospitalization with symptoms lasting >24 hours, or occurring during hospitalization for other reasons. Hospitalization for heart failure was regarded as present when intravenous drug treatment was required for worsening heart failure. Bleeding was adjudicated according to the Bleeding Academic Research Consortium (BARC) classification [3]. BARC type 3 or 5 bleeding was regarded as major bleeding. Any coronary revascularization was defined as either PCI or CABG for any reasons.

**Reference**

[1] Matsuo S, Imai E, Horio M, Yasuda Y, Tomita K, Nitta K, et al. Revised equations for estimated GFR from serum creatinine in Japan. *Am J Kidney Dis*. 2009;53:982-92.

[2] Cutlip DE, Windecker S, Mehran R, Boam A, Cohen DJ, van Es GA, et al. Clinical end points in coronary stent trials: a case for standardized definitions. *Circulation*. 2007;115:2344-51.

[3] Mehran R, Rao SV, Bhatt DL, Gibson CM, Caixeta A, Eikelboom J, et al. Standardized bleeding definitions for cardiovascular clinical trials: a consensus report from the Bleeding Academic Research Consortium. *Circulation*. 2011;123:2736-47.
