# Supplementary material for: Differences in mortality and causes of death between STEMI and NSTEMI in the early and late phases after acute myocardial infarction
Source: PLoS One. 2021 Nov 17;16(11):e0259268. doi: 10.1371/journal.pone.0259268 (PMC8598015; doi:10.1371/journal.pone.0259268)
Supplement: S4 Text — (DOCX) [file pone.0259268.s006.docx]

**S4 Text. List of the clinical event committee members in the CREDO-Kyoto AMI Registry Wave-2**

Masayuki Fuki (Kyoto University Hospital), Eri Toda Kato (Kyoto University Hospital),

Yukiko Matsumura-Nakano (Kyoto University Hospital), Kenji Nakatsuma (Mitsubishi Kyoto Hospital), Hiroki Shiomi (Kyoto University Hospital), Yasuaki Takeji (Kyoto University Hospital), Hidenori Yaku (Mitsubishi Kyoto Hospital), Erika Yamamoto (Kyoto University Hospital), Ko Yamamoto (Kyoto University Hospital), Yugo Yamashita (Kyoto University Hospital), Yusuke Yoshikawa (Kyoto University Hospital), Hiroki Watanabe (Japanese Red Cross Wakayama Medical Center)
