# Supplementary material for: Differences in mortality and causes of death between STEMI and NSTEMI in the early and late phases after acute myocardial infarction
Source: PLoS One. 2021 Nov 17;16(11):e0259268. doi: 10.1371/journal.pone.0259268 (PMC8598015; doi:10.1371/journal.pone.0259268)
Supplement: S5 Text — (DOCX) [file pone.0259268.s007.docx]

**S5 Text. Missing values at baseline characteristics**

There were missing values for body mass index in 146 patients, for LVEF in 649 patients, for eGFR in 15 patients, for hemoglobin level in 13 patients, for platelet count in 19 patients, for Systolic blood pressure in 37 patients, for max CK in 83 patients. The missing values for the risk-adjusting variables were imputed as “normal” in the binary classification, because data should have been available if abnormalities were suspected.
